# General practitioners and general practitioner registrars’ experiences and learning from caring for dying patients: a scoping review

**DOI:** 10.1136/bmjopen-2025-108126

**Published:** 2026-05-03

**Authors:** Rebecca Holdsworth, Hugh Alberti, Bryan Burford, Gillian Vance

**Affiliations:** 1 School of Medicine, Newcastle University, Newcastle upon Tyne, UK; 2 National Institute for Health and Care Research, London, UK

**Keywords:** General Practice, PALLIATIVE CARE, MEDICAL EDUCATION & TRAINING, Health Workforce, Primary Health Care

## Abstract

**Abstract:**

**Background:**

The general practitioners’ (GP) workforce is in crisis, with 22% of GPs feeling so stressed by the pressures of general practice they cannot cope. Patient death is the greatest stressor in medical practice and has an impact on the personal stress and well-being of doctors. Providing good holistic care for dying patients in the community keeps patients in their preferred place of care and reduces unnecessary costly interventions and hospital admissions. It is crucial to explore the experiences and learning of GP and general practitioner registrars’ (GPR)—who represent the future primary care workforce—in caring for dying patients.

**Objectives:**

We aimed to understand what is known about GPs and GPR experiences of, and learning from, caring for dying patients to outline current knowledge and identify future research options.

**Eligibility criteria:**

We included all studies that explored GP and GPR experiences and learning related to adult dying, palliative, terminal care and death.

**Sources of evidence:**

Four electronic databases (MEDLINE, EMBASE, PsycINFO and Cochrane) as well as reference lists and hand-searching key journals were searched from January 2003 until February 2025.

**Charting methods:**

Data were extracted and charted by all authors and then a qualitative content approach was used to analyse and interpret the data.

**Results:**

The database search yielded 3378 publications; 17 studies have been included in the scoping review. This includes over 4412 participants, mostly GPs/GPRs. GPs/GPRs gain knowledge, skills and confidence when they have exposure and hands-on experience with dying patients. Uncertainty, intolerance of ambiguity, fear of initiating conversations around dying and perceived lack of knowledge were barriers for caring for dying patients. Facilitators such as safe learning environments with ongoing support from supervisors and protected time to discuss, debrief and reflect were valuable.

**Conclusion:**

Timely understanding of the current structural, practice level factors such as learning and emotional issues and challenges is required to upskill and support doctors, which can lead to improved emotional well-being and workforce retention—all of which will directly benefit dying patients and relatives at this significant part of their lives.

**STRENGTHS AND LIMITATIONS OF THIS STUDY**

- The scoping review was discussed at different stages including conception, early search strategy, analysis and write-up, with a group of 10 resident doctors, including general practitioner registrars (GPRs) and three qualified GPs.
- ‘VOICE’ a group of public, patients and carers were consulted to gain insight into their ideas and diverse perspectives to ensure the research question and design was acceptable and relevant for more meaningful impact.
- A methodological advantage of this work is the robust search strategy and screening process of four online databases, as well as a broad range of reference lists, hand-searching key journals by the authors.
- Terminology such as end-of-life care, palliative care and care for dying has been used interchangeably in the studies so may not be as specific as the research question intended.
- Initially the objective was to specifically focus on GPRs; however, there was limited literature so the scoping review was broadened to include GPs.

## Introduction

 The majority of palliative and end-of-life care in the UK is provided by generalists and resident doctors, who are not palliative care specialists.^1^ Internationally, the role of general practitioners (GPs) in providing palliative and end-of-life care for patients and their families varies; however, in the UK, GPs play a pivotal role in their care.^2^ GPs are deeply rooted in the community they serve and carry out the bulk of palliative care in the community with support from multidisciplinary teams and specialist providers.^3^ Providing good holistic care in the community keeps patients in their preferred place of care and reduces unnecessary and costly hospital admissions.^4^

Over the last 10–15 years, there have been some significant changes to how care of the dying person is managed in the community and other healthcare settings.^5 6^ An ageing population, who are living longer with more complex needs, means that GP registrars (GPRs) will experience dying and death more frequently in their careers.^7^

GPs and GPRs are encouraged to take the lead and feel comfortable in managing dying and death, but little is known about their experiences, responses to, and learning from, these encounters.^8^ GPs report emotions such as sadness, guilt and anger about patient deaths and traditionally the focus has been on keeping patients alive, with death being seen as a medical failure.^9^ There is limited relevant literature on GPRs specifically but looking more broadly, there is evidence to show that emotions are a major component of resident doctors’ role in caring for dying patients.^10^,^12^ Research shows that resident doctors initially feel unprepared; however, confidence increased as their career progresses and this is influenced by their learning experiences.^10 13 14^ Recognising the learning potential of everyday dying and death experiences and ensuring there is a shift to focus on education and active involvement would support resident doctor learning.

Many GPRs are planning to work less than full time after completing training,^15 16^ while a survey of GPs and GPRs found that 42% said they were likely to leave the profession in the next 5 years because of the intensity of workload pressures.^17 18^ The GP workforce is in crisis, with 22% of GPs feeling so stressed by the pressures of general practice they cannot cope.^19^ Patient death is the greatest stressor in medical practice and has an impact on personal stress and well-being of doctors.^9^ Understanding the experiences of GPs/GPRs and learning from caring for dying people may improve educational strategies, which can support workforce sustainability and benefit dying patients’ and their families’ care.^20^ It is crucial for GPRs to feel confident and skilled to deliver care for dying patients in the community. Therefore, this scoping review aims to identify and map the research on GPR experiences and learning from caring for dying patients to understand the current literature and identify gaps in knowledge.

### Context of study—GP training

GPR is the preferred terminology for doctors in GP training in the UK. GPRs are resident doctors (also known as doctors in training) who have completed medical school and foundation years (or equivalent) before starting 3 years of GP specialist training (ST1/ST2/ST3).^21^ Two of the three specialist training years are completed in primary care (in England and Wales since 2021).^22^

### Palliative care

Palliative care provides relief from pain and other distressing symptoms experienced by people with advanced, progressive illness.^23^ The aim is to support people with active holistic care until death and help their families to cope with the illness and bereavement.^24 25^

### End of life

The General Medical Council (GMC) defines end of life in the context of patients who are likely to die within the next 12 months.^26^

### Dying

Care of patients in the last days and hours of life should include, but is not limited to, the following areas^27^: quality of life, physical symptoms, emotional and cognitive symptoms, advance care planning, functional status, spirituality, grief and bereavement, satisfaction, quality of care and carers’ well-being.

## Methods

This scoping review followed Arksey and O’Malley’s^28^ pragmatic six stages as modified by Levac^29^: (1) identifying the research question, (2) identifying relevant studies, (3) study selection, (4) charting the data, (5) collating, summarising and reporting the results and (6) stakeholder consultation.

### Identifying the research question

The original research question was ‘What are GP registrars’ experiences of, and learning from, caring for dying patients?’. However, this was broadened to include GPs as well as GPRs during the scoping review as outlined in the next stages. ‘Experiences and learning’ are broad terms; however, Arksey and O’Malley suggest a wide definition might reduce the likelihood of missing relevant works and generate breadth of coverage.^28^

### Identifying relevant studies

We conducted a search of electronic databases (MEDLINE, EMBASE, PsycINFO and Cochrane), reference lists and hand-searching key journals. We used a search strategy developed from the research question and definitions of keywords and MeSH terms. Key journals were hand-searched (such as *British Journal of General Practice*, *Family Practice*, *Palliative Medicine*, *Annals of Family Medicine*, *British Medical Journal Supportive & Palliative Care*, *Medical Education Journal*, *BMC Medical Education* and *The Clinical Teacher*) which did not yield any new material. Reference lists and citations were searched which did not identify any further articles.

### Study selection

An iterative approach was used by the team to ensure the process was transparent and replicable to improve rigour.^29^ The screening process was completed by all authors: RH, BB, HA and GV who independently reviewed titles and abstracts. RH reviewed full texts, and any discrepancies were resolved by consensus in a meeting.^29^ There was a very limited amount of research about dying and death, so the search was expanded to include palliative and terminal care, although a focus remained on dying and death learning and experiences. Postgraduate medical training was last re-structured in the UK in 2003 by the GMC,^30^ so January 2003 was chosen as the start date for the search. The search ran until February 2025. There was a lack of specific literature about GPRs involvement so the authors agreed that the search should be expanded to include qualified GPs. Inclusion and exclusion criteria are presented in table 1.

**Table 1:** Inclusion and exclusion criteria for scoping review

Inclusion criteria	Exclusion criteria
From January 2003 (introduction of Modernising Medical Careers) until February 2025	Research focusing on patients under 18 years of age
Any country	Studies of euthanasia and physician-assisted dying, medical aid in dying, palliative sedation, postmortem/autopsy data
English language only	Suicide, sudden or accidental deaths
All research methods	Unpublished manuscripts, conference abstracts, posters, opinion pieces, reviews
Focus on dying and death	Formal education initiatives, eg, course evaluation, curriculum reviews
Adults dying and death	Perspectives from other healthcare professionals or patients, etc
Perspective from general practitioners (GPs), primary care physicians, family physicians, GP trainees’, GP registrars, doctors in training, medical residents in the community	

**Figure 1:**
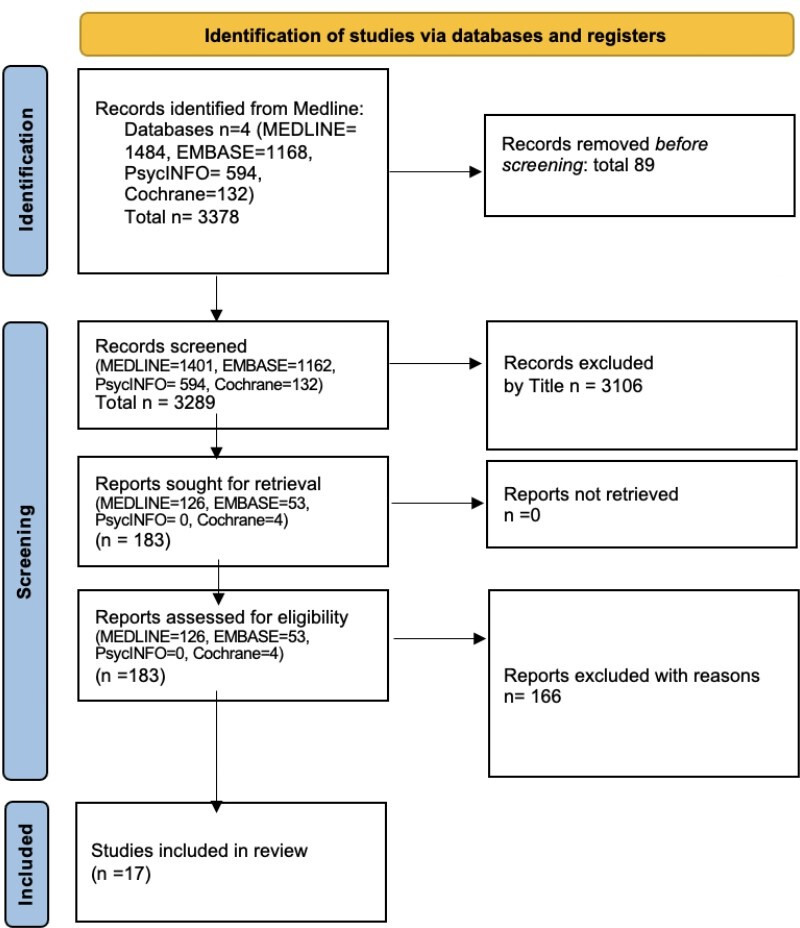
Preferred Reporting Items for Systematic Reviews and Meta-Analyses extension for scoping reviews (PRISMA-ScR)^85^ (see online supplemental file 1).

A Preferred Reporting Items for Systematic Reviews and Meta-Analyses (PRISMA) flow diagram (see figure 1 below) shows the selection steps.

### Charting the data

RH applied a common analytical framework to all primary research reports to collect standard information in Microsoft Excel, including authors, year of publication, study location, intervention type, study population, aims of the study, methodology, outcome measures and important results to inform the analysis.^28^ A detailed table showing the characteristics of studies in the scoping review is available in the online supplemental data.

### Collating, summarising and reporting the results

PRISMA extension for Scoping Reviews checklist was used to report the results. Tables and diagrams presented the narrative of existing literature and identified significant gaps.^28^ An inductive approach was used to create a descriptive framework which was discussed and refined with all researchers.

### Stakeholder consultation

The scoping review was discussed at different stages including conception, early search strategy, analysis and write-up, with a group of 10 resident doctors, including GPRs and three qualified GPs. The stakeholders were identified through personal and institutional contacts. ‘VOICE’, a group of public, patients and carers, was consulted to gain insight into their ideas and diverse perspectives about the research topic and questions. By involving the VOICE public and patient involvement and engagement community, we were able to refine the research question by exploring their lived experiences and identify key issues to improve acceptability, relevance and for the research to have meaningful impact. The role of the doctor group was to analyse data and check the findings were representative of their experiences and learning of dying care.

## Results

The database search yielded 3378 publications, with 3289 screened. Title and abstract screening left 183 publications that were assessed for eligibility. After full text review excluded 165 reports, 17 studies have been included in the review (see table 2).

**Table 2:** Overview of included studies

Participants	Number of studies
Qualified GPs only	5^31 34 39 42 45^
GP training scheme course organisers (non-GPRs)	1^32^
GPs and family physicians/hospital specialists	3^36 38 46^
GPRs only	4^8 33 35 40^
GPs and registered care home nurses	1^32^
Family physician/medicine residents and medical students	2^41 43^
GPs and GPRs	1^44^

Country	Number of studies
Australia	2*^36 45^
Austria	1^37^
Belgium	1*^36^
Brazil	1^41^
Canada	1^43^
Denmark	1*^36^
Germany	1^8^
India	1^46^
Ireland	2^33 34^
Italy	1*^36^
Malta	1^40^
The Netherlands	2*^36 42^
Sweden	1*^36^
Switzerland	1*^36^
UK	5^31 32 35 39 44^
USA	1^38^

Methodology	Number of studies
Qualitative	2^43 44^
Quantitative	11^831 32 34^,^40 45^
Mixed Methods	3^34 41 42^
Systematic review	1^46^

* Part of a multi-centre research study.^36^

GP, general practitioner; GPR, general practitioner registrar.

Over half of the publications (nine studies) were completed between 2003 and 2007,^31^,^39^ two in 2010,^40 41^ one in 2011,^42^ one in 2015,^43^ 2017^44^ and 2020^8^ with two in 2022.^45 46^

### Context of findings: end-of-life care in general practice

The role of the GP being central to providing end-of-life care for patients was widely recognised.^3638 39 42^,^46^ GPs are ‘internally motivated’ to achieve their patients self-actualisation needs and alleviate suffering.^46^ Having a large patient population of elderly and sicker patients increased interest in GPs’ end-of-life care learning.^39 46^ Over four-fifths of GPRs had been involved in the clinical care of palliative patients during their GP training with 73% having a community-based end-of-life experience.^35^ Two studies found GPRs and GPs annually cared for a median of 3–5 terminal patients.^35 36^ One study in Australia found GPs organised and conducted home visits (83.6%), had telephone consultations (77.7%), family meetings (70.5%) and care planning/team-care arrangements (58.6%).^45^

### Main findings from scoping review

**Figure 2:**
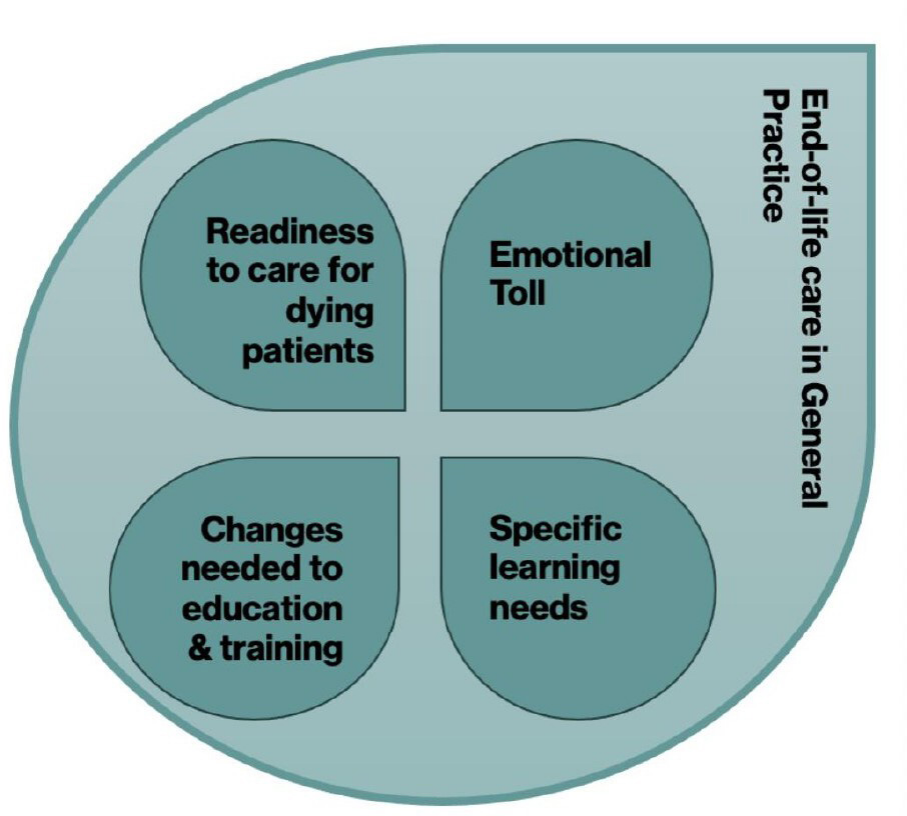
Main findings from scoping review (see online supplemental file 1).

- Readiness to care for dying patients.
- Specific learning needs.
- Emotional toll.
- Changes needed to education and training (see figure 2 below).

### Readiness to care for dying patients

Two factors were associated with perceived increased competency and performance of palliative care: training in palliative care and frequency of providing palliative care.^38^ Physicians who were more interested in palliative care engaged in activities more frequently than those who were uninterested.^38^ Other research consistently found physicians across seven countries with training in palliative care were more likely to discuss palliative care options, including forgoing treatment, than those without training.^36^ One study found that GPs rated their competency higher and educational needs lower, with increasing age, longer professional life and more training in end-of-life care in the 2 years prior to the survey.^37^

GPRs found a lack of exposure to positive role models in community practice disempowered them to incorporate palliative care into their practice perpetuating uncertainty about family physicians’ role in end-of-life care.^43^ End-of-life care was perceived as ‘emotionally and clinically challenging’ by GPRs; however, confidence increased with practical clinical experience with support from a GP trainer.^44^ It was felt that end-of-life care education and experiences during GP training were lost due to lack of exposure and reliance on palliative care specialists or colleagues in GP with a special interest in palliative care.^44^ GPs who were trained more recently felt they had the theoretical knowledge, but training often failed to acknowledge the complexities of real-life practice.^44^

Bereavement was discussed in some papers,^831^,^33 35 46^ with one paper finding less than half reported receiving training in bereavement care and low or very low confidence in their application of knowledge of bereavement care.^35^ Participants in one study felt they needed training for handling the emotional distress of family members.^46^ Patients and families felt valued when doctors recognised family played an important role and they were supported,^33 40^ alongside the patient, with compassion and humility.^41^

### Specific learning needs

Many of the papers discussed pain management as a specific learning need for palliative and dying patients.^31^,^3340 41 46^ One paper stated over three-fifths of GPRs had received training in control of pain and were ‘satisfied’ with 85% feeling moderately confident in applying their knowledge of pain control.^35^ Another paper found pain was highly prevalent in end-of-life care; however, it was classified as ‘well addressed’ by two-thirds of primary care physicians.^38^

Other physical symptoms mentioned in the studies about participants’ formal or informal training, their exposure to specific aspects of care and their self-reported confidence,^31 44^ including control of nausea and vomiting,^32 33 40^ breathlessness,^32 33 40 41^ terminal agitation,^32^ stoma problems,^33^ nutrition,^46^ constipation,^33 40 41^ hypercalcaemia,^33^ delirium,^8^ wound and ulcer care^33^ and insomnia.^41^

Four papers looked at psychological aspects of palliative care for patients,^8 32 45 46^ and three covered depression.^33 40 41^ Four papers discussed the spiritual aspects of care of the dying,^8 32 38 46^ with one paper finding half of the participants perceived they addressed the spiritual needs of the patient in a ‘fair’ or ‘poor’ manner,^38^ and a systematic review found doctors identified religious understanding as a learning need for end-of-life care.^46^ Two papers reviewed financial issues^32 38^; in one study, participants found that they did this in a ‘fair’ or ‘poor’ manner.^38^ One paper found just over half the primary care physicians (defined as internal medicine and family practice physicians) caring for patients at the end-of-life care reviewed financial needs, spiritual needs and considered different cultures of patients.^38^ Some papers discussed ethical issues,^32 33 40^ including questions of euthanasia.^32 33^ One paper found that GPs wanted to learn how to discuss ethical and medico-legal aspects of care with their patients, families and colleagues.^46^ These aspects are important to address in end-of-life care but are lacking in the research found in the scoping review.

The value of and challenges of communication were recognised in the studies, particularly the difficulty of breaking bad news,^33 40 46^ general communication skills with dying patients and relatives^32 33 40^ and the ability to detect real needs by listening.^41^ Training and learning needs were identified for GPRs around communication,^35 44 46^ and one discussed the lack of confidence in this area.^36^ One paper found that GPRs felt communication skills were covered well during training; however, it was an ongoing challenge to initiate discussions of palliative care and providing emotional support to families.^44^ On the contrary, one paper reported only half of GPRs received training in communication skills, but 86% were moderately confident in applying their knowledge.^35^ Importantly, one paper found those with training in communication skills were more comfortable discussing palliative care options and forgoing potential life-sustaining treatments than doctors without training.^36^ GPs were apprehensive about initiating discussions around end-of-life, particularly spirituality, as this was perceived as a taboo by patients and families.^46^ The same study found there was a general fear of medico-legal recrimination.^46^ One paper found participants struggled to ‘find the right words’ and communicate effectively,^40^ while others felt they needed specialist learning to deal with anger and demanding behaviour.^46^ Another study found a barrier for caring for dying patients was resistance among patients to discuss death or dying, as well as doctors’ fear about justifying decisions to not refer patients to hospital or to stopping medications.^44^ Participants in another study felt that advanced training could support them to have diplomacy to deal with ‘conflict, denial, bargaining and vulnerability around dying and death’.^46^

### Emotional toll

GPRs admitted that dealing with their own emotions was a significant source of stress, with a third feeling their medical training had not given them the necessary skills to cope.^33^ A systematic review mentions fear of dying and death was high among doctors as an educational and learning need.^46^ GPRs state that the emotional stress was highest during the final hours of a patient’s life but breaking bad news, time pressures and dealing with uncertainty around prognosis were also sources of stress.^33^ This was echoed by other studies; many GPRs worrying about managing future patients and dying patients at home.^40^

GPRs worried about managing their self-care^40^ and some self-actualisation needs, such as integrating palliative care into routine care, coping with their own bereavement and perceived inability to manage symptoms, left GPRs feeling powerless,^44^ helpless^44^ and emotionally burdened from caring.^46^

There was an equal divide between GPs feeling ‘competent’ and ‘not competent’ for their ability to cope with distress.^37^ GPs felt debriefing sessions to cope with emotionally burdensome situations would be helpful.^46^ In another study, 40% of GPRs were fearful of managing dying patients.^40^ The study suggests that there may be inadequate preparation for the reality of clinical practice at all phases of life, including dying.^40^ Many barriers have been reported by GPs to providing good palliative and dying patient care including dealing with patient fears and strong emotions, not knowing patients’ wishes and expectations, a lack of continuity of care and high demands from patients relatives^42^ and the emotional impact of care on the doctors.^46^ Although GPs preferred to avoid feedback from dying patients and families as they felt it could cause distress,^46^ feedback from patients they cared for in the final week of their lives gave GPs high satisfaction.^45^

Teamwork was regularly mentioned as essential for support and well-being for the GPs and GPRs in the research in palliative care and caring for dying patients.^33 40 43 46^ Timing the referral^43 46^ and involving specific multidisciplinary team members such as palliative care specialists were challenging but vital, particularly when patients were transitioning from stable to imminently dying or the patients’ needs were complex.^43^

### Changes needed to education and training

Irrespective of earlier palliative care training, many physician respondents (between 57% and 98%) wanted extended training in end-of-life care^33 35 36 43 44^ as GPs and GPRs felt inadequately prepared.^35 37^ One paper found that the median hours of formal teaching of palliative care on a 3-year GP training programme was 9 hours and 4.75 on a 1-year training programme.^32^ Regardless of the training method, GPs felt the learning environment had to be safe, non-judgemental, trustworthy, respectful and not exposing to their patients.^46^

GPs and GPRs recognised that opportunistic training could result in inconsistent, inadequate exposure and education in end-of-life care in undergraduate and GP training.^44^ There was a sense that a lack of real-life experience impacted on confidence and opportunity to improve skills despite having the theoretical knowledge.^35^ Lack of time to provide good palliative care was a large barrier discussed in the research.^42^ GPs who work in rural areas or solo practices had fewer support systems with excessive work pressures and poor remuneration and often struggled to engage in training.^34 46^ GPs with personal or family commitments had less time for palliative care provisions.^34 46^ There was a lack of knowledge of local services, such as district nursing support and specialist palliative care services out of hours.^39^

Opinions differed about the most effective way to deliver teaching, and what stage of training would be best, for palliative care.^36 46^ A mixture of teaching methods were used for palliative care training in the literature, such as lectures,^32 46^ seminars,^32 34^ role play,^32 44^ simulated patients,^32^ clinical case discussions,^32 44^ journal writing,^41^ mentorship^44^ and hospice visits or placements.^32 33 35 44^ Real-life experience was considered the gold standard by GPRs and qualified GPs, ‘No more lectures…we need experience’*.*^44^ Tutorials given by any doctor or trainer or half-day protected teaching time was preferred methods over placements in hospital settings for GPRs.^40^ Respondents wanted training to be integrated into their clinical practice and existing meetings.^44^ GPs accessed training if they perceived the training and the trainers’ skills aligned with their needs and addressed complex end-of-life care issues.^46^ Peer support through mentorship was suggested as a way of acknowledging challenging situations and offering support and advice.^44 46^ Experience in hospices with palliative specialists was valuable^44^ but many GPRs have not had this opportunity.^33 35^

E-learning had mixed responses: some felt it was not appropriate for palliative care,^44^ required a significant time commitment,^34^ was difficult to absorb and retain the information,^44^ whereas some GPs preferred self-directed learning to other methods^46^ as it is flexible and cost-effective.^44^

Experiences and learning prior to GP specialist training, as medical students and resident doctors, were detailed in a few studies as being undervalued and needing improvement.^8 33 34^

Early exposure as medical students to experiences and teaching between hospitals and GP could improve palliative care training of medical students and GPRs.^8^ Prior to starting GP training, some GPRs had completed specific palliative care placements (10%), while 6% had trained in a medical specialty.^33^

## Discussion

This scoping review highlights the vital need to acknowledge the multi-factorial facilitators and barriers and suggests implications for training reform for GP/GPR experiences and learning from caring for dying patients. For dying people and their relatives, individualised, compassionate and dignified care are fundamental, which is why it is so important to act on the factors identified to improve understanding, training and learning.

### Practice level factors

A variety of factors facilitate positive interactions, but some barriers prevent doctors from providing attentive holistic care for dying patients. GPs/GPRs benefit from positive role models,^47^ GPs prefer to coordinate dying patients ongoing care^48^,^50^ but value facilitatory support from specialists.^48^ Supportive ‘real life’ learning environments with protected time for teaching, feedback and reflection^51^ are crucial for learning end-of-life care.^52^ Longitudinal relationships between role models and learners improve trust and openness which facilitates learning on many levels.^51^ It can improve well-being and reduce burnout by role models showing self-awareness and demonstrating positive coping strategies such as willingness to resolve problems, boundary setting and dealing with stressful situations.^51 53^ Uncertainty has been recognised by healthcare professionals as a significant barrier to providing good palliative care and recognising dying.^54^ Improving uncertainty tolerance, defined as adaptively responding to perceived uncertainty, benefits health practitioner well-being and improves person-centred care. Lower uncertainty tolerance is linked to adverse outcomes.^51^ Building a range of clinical experiences can support learning to distinguish known unknowns from unknown unknowns and encourage learning from ambiguous situations.^51^ Uncertainty may provoke unpleasant emotions and reflection, using a framework such as ‘what, so what, now what?’ allows distance from the emotions to instead focus on the learning benefits.^51 55 56^

### Structural factors

With the introduction of the NHS 10-year health plan for England^57^ and the Terminally Ill Adults (End-of-life) Bill^58^ under review at the House of Lords, NHS healthcare is arguably experiencing the biggest changes since its inception. There are currently numerous unmet needs in end-of-life care in the community, with an increasing number of unidentified non-cancer end-of-life patients dying without the services and support they require.^54^ The 10-year health plan aims to shift from secondary care to delivering services in the community and at patients’ homes, with emphasis on improved access to integrated palliative care, supporting patients choices including preferred place of death, agreed care plans for complex patients and more holistic support, while addressing workforce challenges for palliative care.^59^

With the day-to-day challenges of modern healthcare,^60^ lack of continuity for patients,^49 57^ higher demands and unrealised patient and family expectations^61^ and more complex patients needs,^60 62^ the increased pressure on GP/GPR workload^49 62^ can have an impact on caring for dying patients. Other barriers include lack of time^63^ and poor understanding of, and limited local resources and services.^60 64^ There are practical difficulties when delivering care for dying patients which can be deflating for healthcare professionals, patients’ and families, such as when deaths in preferred places of care cannot be facilitated due to resources and funding. The literature shows that holistic,^65^ patient-centred care^66^ with continuous patient-doctor relationships^63^ and increased time with patients^50 64^ improve dying experiences for patients and families.^65^ It allows everyone to feel involved and less fearful and more satisfied, as having the opportunity to discuss dying and feel listened to benefits family and their ability to support patients.^47 63 67^ This in turn allows doctors to feel more positive about the experience^68^ and build on this for future patients care, which is so important for healthcare workers and patients needing future dying care.

### Implications for training reform

Training reform is essential to improve GPRs’ knowledge, skills and comfort to deliver good quality dying care for patients. This scoping review recommends more frequent clinical encounters with dying patients for all GPRs and ‘hands-on’ experience^69^ with support that is acceptable for GPR learning and emotional needs. If these core principles can be implemented consistently within GP training, GPRs can improve their knowledge, practical skills and contentment in discussing end-of-life and dying options with patients and families including best supportive care, where appropriate. These training reforms will avoid unwarranted interventions for dying patients,^70^ unnecessary hospital admissions,^71^ cause less panic^72^ and provide better experiences for patients.^71^

A key focus for GPR training needs to be around initiating conversations about dying with patients and relatives,^63 72 73^ navigating uncertainty and being tolerant of ambiguity around end-of-life care.^54 55^ Home visits^48 57 65^ can provide the time and space for these interactions and support subsequent difficult conversations and encounters. Continuous mentoring, peer support and experiential learning from GPs, GP supervisors and specialists are more valued than attending formal training.^44^

Frequent opportunities that are equitable to all GPRs, not just ad-hoc encounters for those with a special interest, are useful. The GPRs need support with managing these experiences and protected time for debriefing with colleagues.^64 74^ A priority training reform outcome would be for GPRs to progressively become more independent in delivering dying patient care as they gain clinical experience through their training, while simultaneously receiving a decreasing level of clinical supervision.^75^ GPs/GPRs can be insecure at actioning their areas of low clinical confidence and can lack insight into engaging in educational activities to improve their skills, instead choosing to focus on other areas of interest and higher confidence.^8 76^ If this issue is not addressed, it could have a detrimental cascade effect for GPR training and the future workforce; perpetuate a downward spiral of end-of-life care provided in the community.^64 68^

An area requiring training reform is offering GPRs routine support and learning methods to manage their own and their patients bereavement and after-death care for relatives.^66 77^ Positive dying encounters can be rewarding^47 50 68^ for GPRs and can act as a catalyst for further fulfilling experiences, leading to increased confidence and self-reported competencies.^49^ Identifying and supporting GPRs who may feel stressed and overwhelmed about delivering dying patient care can improve emotional well-being, reduce emotional burden and burnout,^68 77^ which in turn helps with GP retention and primary care workforce planning.

### Limitations of previous work

This scoping review has identified numerous gaps in the literature around the timing of the studies, as well as the type and depth of the studies. Over half of the studies were completed before 2010 and practice, public engagement and education have all changed significantly over that time.^62^ For example, there is now less emphasis on GPs setting up syringe drivers,^78^ a decline in single-handed GP surgeries in the UK,^79^ as well as an increase in allied health professionals^80^ including specialist community roles, more access to internet, intranet and telephone advice^81^ and collaborative Palliative Care and General Practice guidelines to help with best practice and service navigation in end-of-life care.^6 82^

Many of the studies within the scoping review focus on confidence and competency through self-rated questionnaires. Evidence has shown that physicians have limited ability to effectively identify areas of weakness and accurately self-assess gaps in competence.^76 83^ It could be argued that this is not an appropriate assessment of knowledge, competency and educational needs.

Many of the surveys published mention one brief statement about each aspect of dying care which leads to a lack of depth of understanding about why or what the participant is thinking and the significance of this finding. Spiritual needs, financial support and medico-legal aspects of care seem to be poorly addressed in the literature but should be included in care for dying patients routinely by GPs/GPRs.^65 69 84^

Other limitations of included studies are the lack of literature about GPRs specifically, which is why this scoping review included qualified GPs. There are many nuances between end-of-life care, palliative care and care for dying people and the terminology has been used interchangeably in the studies. There is very little specifically about the care of dying patients, there is more focus on end-of-life or palliative care. There is little to no mention of organisational or management systems within this scoping review data and this would be helpful to review and assess in future research.

### Implications

This scoping review, the first globally on this topic, has identified a number of factors that require urgent medical educational review and training reform. Research gaps have been identified, including a limited focus on GPRs specifically; minimal up-to-date research in the context of large policy and social changes in dying care in the last 10 years and a reliance on self-questionnaires which lack depth and would not be appropriate for suggesting implementation reform to current care for dying patients.

Future research is required to deepen the understanding of the current context for GPs and GPRs and explore the quality of experiences, learning and barriers around caring for dying patients. This, in turn, can support effective planning of practical measures, policies and educational theory. Work is needed to establish the most effective way of implementing changes that have a short- and long-term effect at a regional and national level.

Key findings suggest hands-on experiences with supportive supervisors and protected time for clinical discussion, debrief and reflection are essential; but we need to understand how we can safeguard these facilitatory factors and resources for learning, while reducing the impact of daily inhibitory barriers within the healthcare setting.

## Conclusion

This review considers GP and GPR experiences and learning in caring for dying patients. Our results have identified an urgent need for development of medical education in caring for dying patients and end-of-life care for GPR. By addressing the current structural, practice level issues and challenges, doctors can be upskilled and supported, leading to improved emotional well-being and retention—all of which will directly benefit dying patients and relatives at this crucial part of their lives. Improving training will build a positive foundation and a springboard effect for GPRs who become the GPs of the future, and role models for more fulfilling care for dying patients. Future research is needed to gain a deeper understanding of the GPR experiences and learning needs for caring for dying patients, to develop innovative educational practice, theory and policy for long-term dying patient care strategies.

## Supplementary material

10.1136/bmjopen-2025-108126 online supplemental file 1

10.1136/bmjopen-2025-108126 online supplemental file 2

## Data Availability

No data are available.
