# Supplementary material for: General practitioners and general practitioner registrars’ experiences and learning from caring for dying patients: a scoping review
Source: BMJ Open. 2026 May 3;16(4):e108126. doi: 10.1136/bmjopen-2025-108126 (PMC13141103; doi:10.1136/bmjopen-2025-108126)
Supplement: online supplemental file 2 [file bmjopen-16-4-s002.docx]

Supplementary File:

Embase search terms


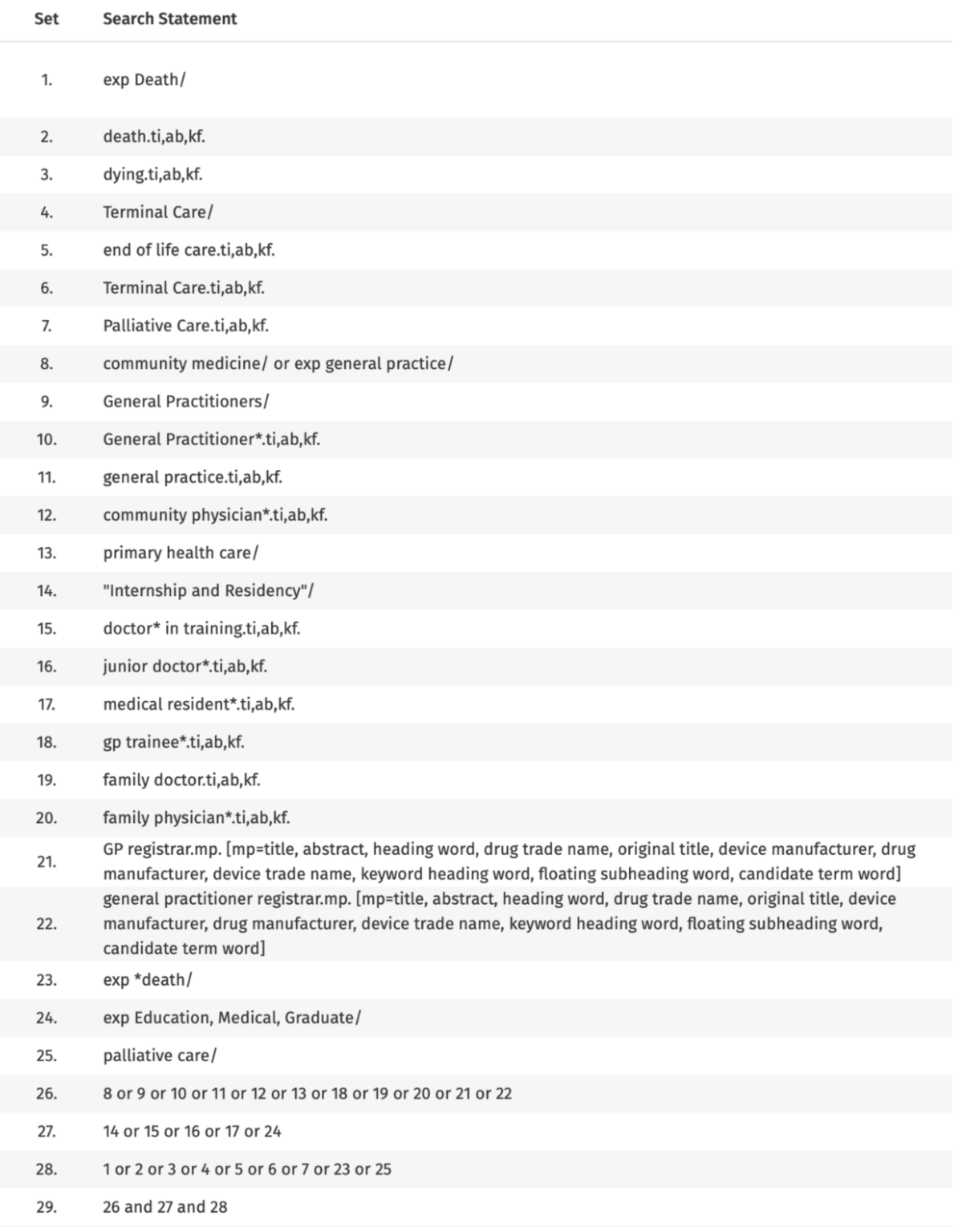


PsycINFO search terms


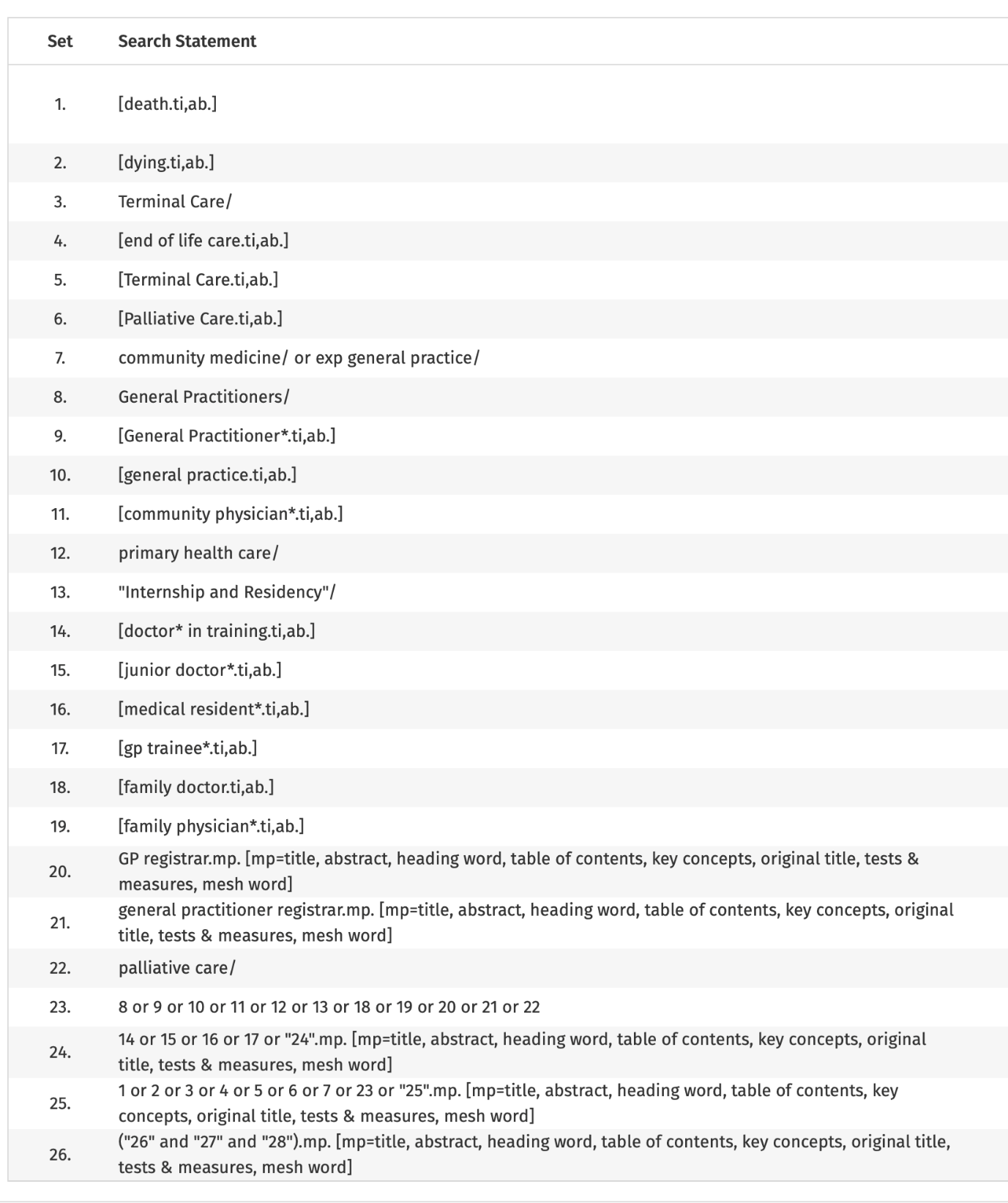


Medline search terms


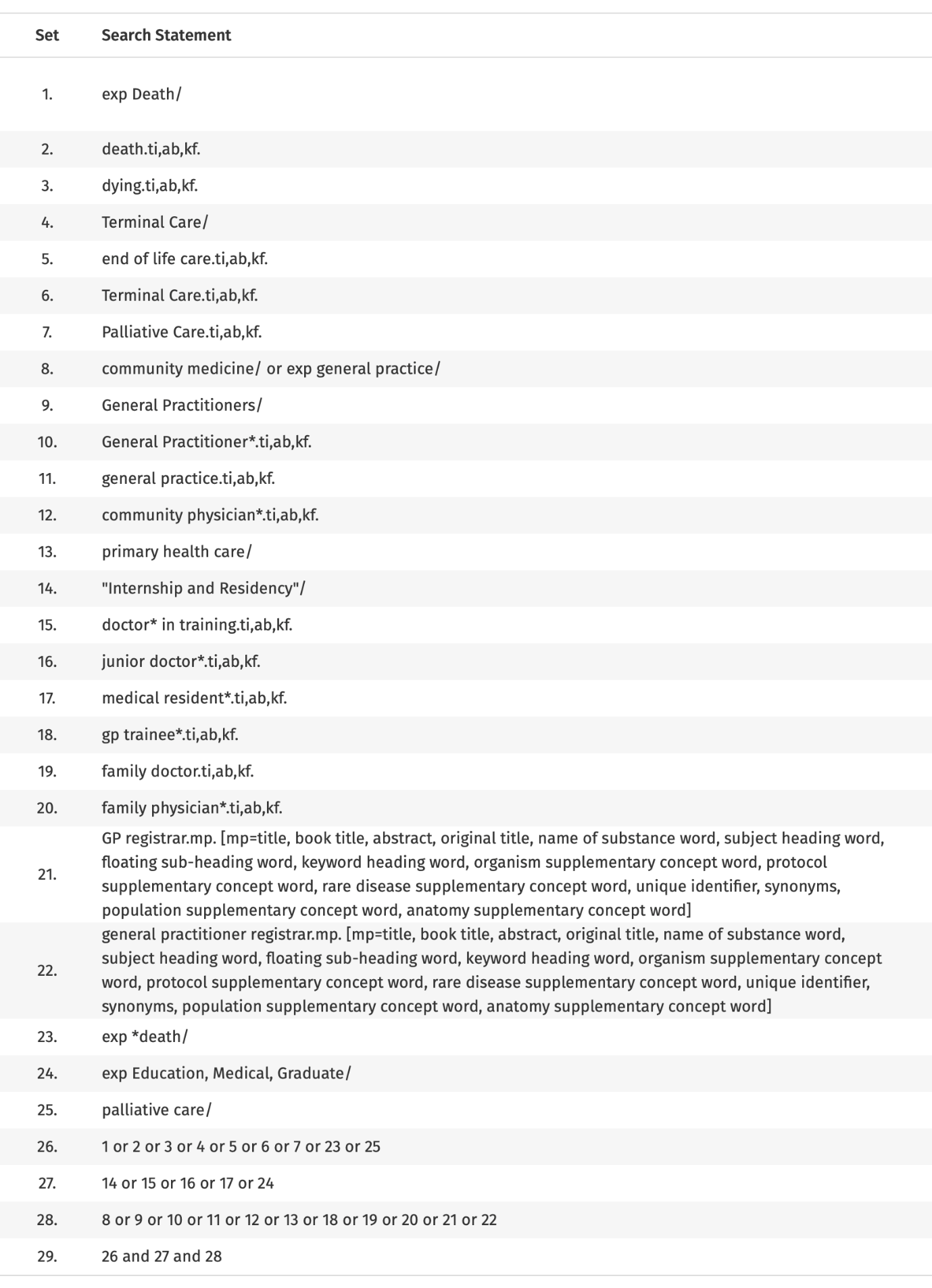


Cochrane Library search


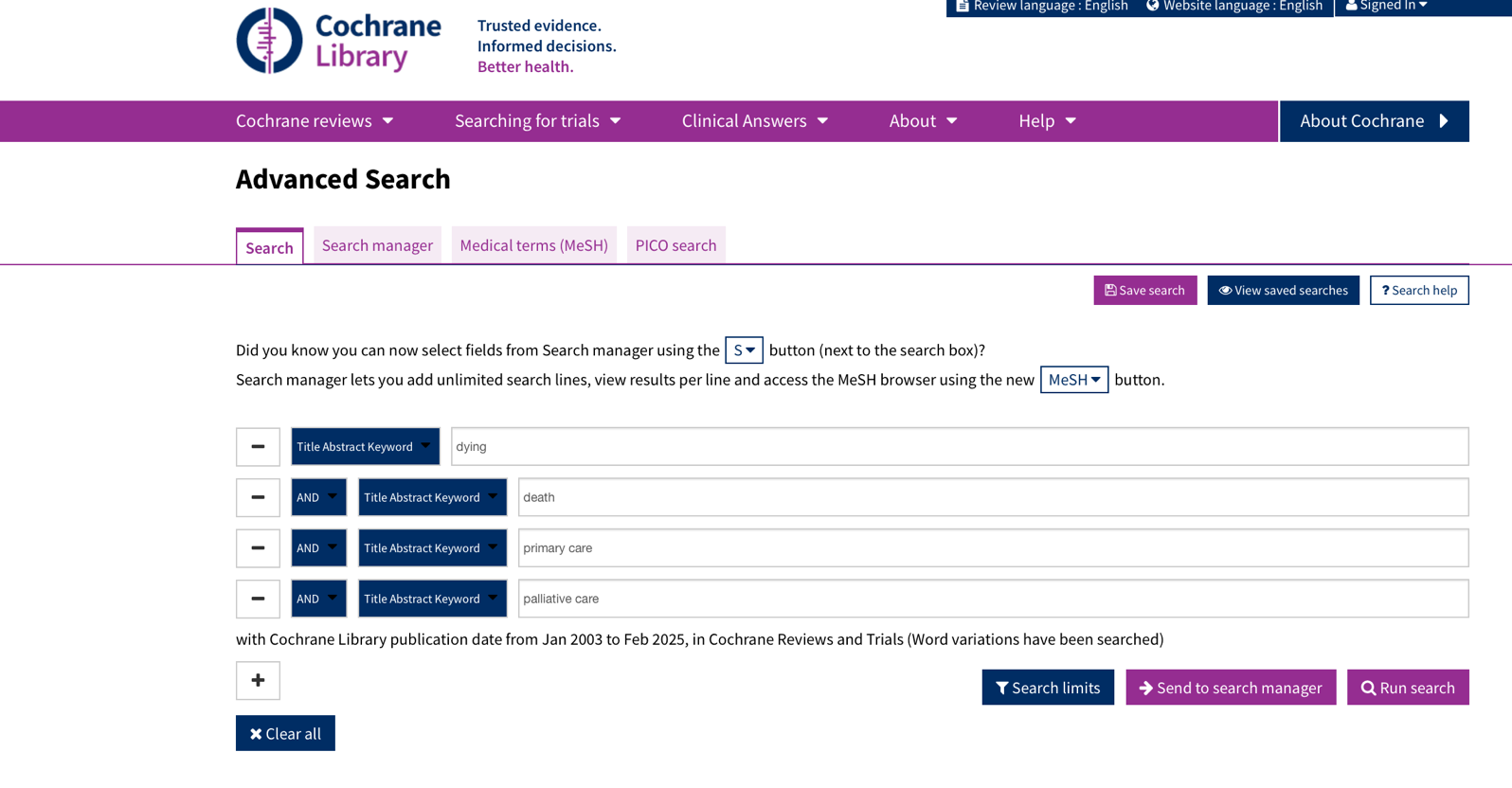


**Table 2:  Details of each study including year of publication, role, stage of training and number of participants, location, response rate, type of study and demographics**

| Title & Reference | Year of publication | Role, stage of training and number of participants in study | Location of study | Response Rate | Type of study | Demographics (all details given) |
| --- | --- | --- | --- | --- | --- | --- |
| 1 Education in palliative care: A questionnaire survey of Irish general practitioner trainees | 2005 | 51 final year GP registrars | Ireland | 94% | Questionnaire/survey | Mean age 29 years old, Male 31%, Female 69%, All Irish universities graduates. Practice attachments - urban 43%, rural 33% mixed 24%The study subjects worked in urban, rural and mixed practices; the average number of doctors working in each practice, including the GP registrar, was four. The majority of GP registrars had previous experience in a variety of medical and surgical specialties including medicine 100%, A&E 64.7%, paediatrics 96%, Psychiatry 84%, Obstetrics and gynaecology 58%, Eyes/Ear, Nose Throat 21%. Involvement in or experienced the death of a palliative care patient- medical student 37%, junior doctor 92%, GP registrar 72%. Number of patients that respondents provided palliative care for in the last 12 months- none 23%, 1 23%, 2 21%, 3 16% 4-8 16%. |
| 2 General practice vocational training in the UK: what teaching is given in palliative care? | 2003 | 240 GP tutors on the GP vocational training scheme | UK | 64% | Questionnaire/survey | Vocational training scheme tutors for both one-year and three-year scheme. Thirty-one (13%) course organizers were responsible for one-year schemes; 95 (40%) for three-year schemes and 102 were responsible for both one-year and three-year schemes (42%) data was missing for 12 (5%) |
| 3 Caring for the dying: how well prepared are general practitioners? A questionnaire study in Wales | 2003 | 399 GPs- GPs asked to recall training medical student🡪 hospital doctor 🡪GP registrar🡪 GP | UK | 67.6% | Questionnaire/survey | Gender M= 71.4%, F=28.6%, Mean age 43.8 years old, medical school (London 19%, Cardiff 39.6%, non-UK 14%, rest of UK 27.3%), mean years qualified- 19.9 years, trainee year yes=84%, no=64%, mean years as GP principle= 12.5 years, M/FRCGP yes 72.2%, no 27.8%, training practice yes 39.5% no 60.7%, trainer yes 14% no 86%, mean number of partners=4.5 |
| 4 A UK-wide postal survey to evaluate palliative care education amongst General Practice Registrars | 2006 | 320 GP registrars | UK | 65% | Questionnaire/survey | mainly female (61%), median age 30 years; 80% had qualified within the last 10 years; 51% were on a three-year programme with 46% on self- construct programme. All had been working as GPRs for a median of 11 months. |
| 5 Competency and educational needs in palliative care | 2007 | Overall, 546, GP only 228/897 | Austria | Overall, 30%, GP 25% | Questionnaire/survey | GPs median years of experience = 18 years with a range from 1 to 52 years. Female 22.4%, male 77.6%, mean age 47.1, PC training in the last two years- 78.6%, no training 21.4% |
| 6 Palliative care training: a survey of physicians in Australia and Europe | 2006 | Multiple medical specialties, maximum GPs=300 from each country | Australia, Belgium, Denmark, Italy, Netherlands, Sweden and Switzerland | Range from 35% to 78% | Questionnaire/survey | Undergraduate training was most frequent in Australia and Sweden, and postgraduate training most common is Belgium, Denmark and Netherlands. The most common training was a postgraduate course.  There were no variations according to sex and age except that Australian physician younger than 40 years old had more frequently had palliative care training than Australian physicians older than 50 years old (62% versus 35%). Physicians in nursing home medicine, geriatrics, oncology and general practice had had most training, while those in gynaecology, neurology and surgery had had the least. In Australia, physicians were trained for relatively more days than in other countries. Five percent to 22% of physicians had not been responsible for any terminal patients during the last year, while between 6% and 12% had cared for more than 20 patients. The median number of patients was similar (3-6) regardless of any palliative care training. |
| 7 An evaluation of palliative care education in the specialist training programme in family medicine | 2010 | 22 GP registrars | Malta | 74.4% | Questionnaire/survey | 14 female GP trainees and 8 male GP trainees |
| 8 Facilitators and barriers for GP-patient communication in palliative care: A qualitative study among GPs, patients, and end-of-life consultants | 2011 | 20 GPs | Netherlands | - | Focus groups, interviews and questionnaires | GPs=20 M=13, f=7, Mean age 49.5 years old. Mean clinical work experience 17.7 years. Urban =14, semi-rural=6, trainers=10, non-trainers=10, very experienced in PC=6, no=14 |
| 9 Are family medicine residents adequately trained to deliver palliative care? | 2015 | 15 family medicine Residents | Canada | - | Interviews | N=9-first year, N= 6 second year, first palliative care experience for 9, 8 had previous experience during medical school. |
| 10 How to ensure basic competencies in end of life care- A mixed methods study with post-graduate trainees in primary care in Germany | 2020 | Between 75-219 GP registrars for different questionnaires | Germany | Range 40.9- 98.6% | Survey including short answers and space for personal reflection | Group I were mostly female (*n* = 143/219, 65.3%). In group C, 76% (*n* = 57/75) were female. Mean age was 36.6 years (SD 6.8) in group I in comparison with 36.7 years (SD 6.9) in group C (*p* = 0.74.) |
| 11 Primary care physicians' educational needs and learning preferences in end of life care: A focus group study in the UK | 2017 | 10 qualified GPs and 18 GP registrars | UK | - | Focus groups | Participants comprised 18 trainees (8 ST1s, 3 ST2s, 7 ST3s) and 10 GPs (median years in practice 9, range 3-45). The majority were women (79%), with a median age of 32 (range 27-63) ^45^ white British 16, Sri Lankan 1, white and black African 1, Pakistani 3, black African 2, Indian 3, other mixed/multiple 1, not disclosed 1.^45^ |
| 12 Frequency and perceived competence in providing palliative care to terminally ill patients: A survey of primary care physicians | 2004 | 463 of internists and family medicine physicians | America | 48% | Questionnaire/survey | The responding physicians had an average age of 46 years and were largely male and married. While a majority of respondents had experienced a life-threatening illness in a spouse or loved one, only 12% had personally experienced such an illness. The practices of most respondents were in urban or suburban settings, and most were in private practices. Physicians spent an average of 87% of their time seeing patients. On average, the respondents' practices consisted of only 6% terminally ill patients, with respondents caring for only 19 patients per year with a terminal illness. Respondent physicians provided palliative care to an average of 67% of their terminally ill patients. Only 36% of the respondents had received any type of training in palliative care. |
| 13 Provision of end-of-life care in primary care: A survey of issues and outcomes in the Australian context | 2022 | 63 GPs reviewing 220 expected deaths | Australia | 9.5% | Questionnaire/survey | More GPs were male (55.5%), and between 50 and 59 years old (38.1%). Approximately half were born in Australia (54.0%) and practised in regional or rural/remote areas (54.0%). The majority received primary medical training in Australia (74.6%). On average, participants had 23 years of work experience and worked 40.7 hours per week. Less than one-third of GPs had ever received formal palliative care training (30.1%). They seldom used symptom assessment tools (11.1%). |
| 14 Roles, service knowledge and priorities in the provision of palliative care: A postal survey of London GPs | 2006 | 356 GPs | UK | 57% | Questionnaire/survey | Female GPs were slightly over-represented (58% of our respondents versus 46% in London as a whole and 39% in England. Single-handed GPs were under-represented (6% of our respondents versus 18% in London and 9% in England. A lower-than-expected proportion of GPs aged 55 and over responded, compared to London GPs as a whole (21 versus 29%), although there were no significant differences by age compared to GPs in England (20%).  Most GPs (65%) reported that they were currently providing palliative care to patients on their list: 61% to cancer patients, and 26% to non-cancer patients. Overall, most (63%) were currently caring for between one and three patients, 22% for four to six patients, and 15% (32/211) for seven or more. |
| 15 Views of general practitioners on end-of-life care learning preferences: a systematic review | 2022 | 10,037 articles identified in systematic review, 23 included | India | - | Systematic review | General practitioners or family physicians |
| 16 Palliative care education: A Delphi survey of Irish general practitioners | 2005 | 32 GPs | Ireland | 40% | Delphi survey | Eighteen (56%) were male; 70% had been working as GPs for ten years or more. The majority were working in a mixed practice (surgeries in both urban and rural settings); all but two worked full time. Just under two-thirds of GPs had cared for between three and six palliative care patients in the previous year. One GP had seen no such patients in the previous 12 months. Many of those surveyed had completed a postgraduate course in palliative care. |
| 17 Teaching palliative care to residents and medical students | 2010 | - | Brazil | - | Combined journal entries, questionnaire and reflective papers | Only demographics given were that the participants were family physicians residents and medical students. |

References

1. Dowling S, Leary A, Broomfield D. Education in palliative care: A questionnaire survey of Irish general practitioner trainees. Education for Primary Care. 2005;16(1):42-50.

2. Lloyd-Williams M, Carter YH. General practice vocational training in the UK: what teaching is given in palliative care? Palliat Med. 2003;17(7):616-20.

3. Barclay S, Wyatt P, Shore S, Finlay I, Grande G, Todd C. Caring for the dying: how well prepared are general practitioners? A questionnaire study in Wales. Palliat Med. 2003;17(1):27-39.

4. Low J, Cloherty M, Wilkinson S, Barclay S, Hibble A. A UK-wide postal survey to evaluate palliative care education amongst General Practice Registrars. Palliat Med. 2006;20(4):463-9.

5. Becker G, Momm F, Gigl A, Wagner B, Baumgartner J. Competency and educational needs in palliative care. Wiener Klinische Wochenschrift. 2007;119(3-4):112-6.

6. Lofmark R, Mortier F, Nilstun T, Bosshard G, Cartwright C, Van Der Heide A, et al. Palliative care training: a survey of physicians in Australia and Europe. Journal of Palliative Care. 2006;22(2):105-10.

7. Abela J, Mallia P. An evaluation of palliative care education in the specialist training programme in family medicine. Malta Medical Journal. 2010;22(4):26-33.

8. Slort W, Blankenstein AH, Deliens L, Van Der Horst HE. Facilitators and barriers for GP-patient communication in palliative care: A qualitative study among GPs, patients, and end-of-life consultants. British Journal of General Practice. 2011;61(585):e167-e72.

9. Mahtani R, Kurahashi AM, Buchman S, Webster F, Husain A, Goldman R. Are family medicine residents adequately trained to deliver palliative care? Canadian Family Physician. 2015;61(12):e577-82.

10. Schwill S, Reith D, Walter T, Engeser P, Wensing M, Flum E, et al. How to ensure basic competencies in end of life care- A mixed methods study with post-graduate trainees in primary care in Germany. BMC Palliative Care. 2020;19(1).

11. Selman LE, Brighton LJ, Robinson V, George R, Khan SA, Burman R, et al. Primary care physicians' educational needs and learning preferences in end of life care: A focus group study in the UK. BMC Palliative Care. 2017;16(1):17.

12. Farber NJ, Urban SY, Collier VU, Metzger M, Weiner J, Boyer EG. Frequency and perceived competence in providing palliative care to terminally ill patients: A survey of primary care physicians. Journal of Pain and Symptom Management. 2004;28(4):364-72.

13. Ding J, Johnson CE, Saunders C, Licqurish S, Chua D, Mitchell G, et al. Provision of end-of-life care in primary care: A survey of issues and outcomes in the Australian context. BMJ Open. 2022;12(1).

14. Burt J, Shipman C, White P, Addington-Hall J. Roles, service knowledge and priorities in the provision of palliative care: A postal survey of London GPs. Palliat Med. 2006;20(5):487-92.

15. Atreya S, Datta SS, Salins N. Views of general practitioners on end-of-life care learning preferences: a systematic review. BMC Palliative Care. 2022;21(1).

16. Dowling S, Leary A, Broomfield D. Palliative care education: A Delphi survey of Irish general practitioners. Education for Primary Care. 2005;16(4):458-66.

17. Pinheiro TR, De Benedetto MA, Levites MR, Giglio AD, Blasco PG. Teaching palliative care to residents and medical students. Family Medicine. 2010;42(8):580-2.
