# Supplementary material for: General practitioners and general practitioner registrars’ experiences and learning from caring for dying patients: a scoping review
Source: BMJ Open. 2026 May 3;16(4):e108126. doi: 10.1136/bmjopen-2025-108126 (PMC13141103; doi:10.1136/bmjopen-2025-108126)
Supplement: Supplementary file 3 [file bmjopen-2025-108126.reviewer_comments.pdf]

PEER REVIEW HISTORY

BMJ Open publishes all reviews undertaken for accepted manuscripts. Reviewers are asked to complete a checklist review form and are provided with free text boxes to elaborate on their assessment. These free text comments are reproduced below.

ARTICLE DETAILS

Title (Provisional)

General Practitioners and General Practice registrars experiences and learning from caring for dying patients: A scoping review

Authors

Holdsworth, Rebecca; Alberti, Hugh; Burford, Bryan; Vance, Gillian

---

VERSION 1 - REVIEW

**Reviewer**	**1**
**Name**	**Pai, Radhika R**
**Affiliation**	**Manipal College of Nursing, Fundamentals of Nursing**
**Date**	**04-Nov-2025**
**COI**	**None**

Please cosider responding to following querries for Improvement

Abstract:

For clarity, think about dividing it into organized subheadings (Background, Objectives, Methods, Results, Conclusion).

Introduction

For easier reading, several lengthy sentences might be divided.

For instance: "Offering excellent, comprehensive care to patients nearing the end of their lives in the community keeps patients in the setting of their choice and lowers the need for needless, expensive interventions and hospital stays." → For clarification, it might be divided into two sentences.

The phrases "end-of-life," "palliative," and "dying" are used interchangeably. Think about defining them in advance and using them consistently all the way through.

Methods

It would be more accurate to say that "Ethical approval was not sought" instead of "Ethical approval was not sort."

For improved reading, format the inclusion/exclusion table in the final version as a proper table.

Make sure the additional appendices for search terms you mention is comprehensive and easily accessible.

Results

Although it is not evident in the text, the PRISMA-ScR diagram is mentioned. Make sure the final submission contains it.

To make the four primary topics more visually identifiable, think about utilizing bullet points or subheadings.

Table 2: For easy access, think about incorporating a summary table into the main text (rather than only supplementary).

Discussion

The conversation is thorough and properly cited. Nonetheless, it would be advantageous to distinguish more clearly between: structural/systemic problems, learning/emotional variables, Consequences for training reform

Certain ideas are reiterated, such as the value of practical experience and mentorship. For conciseness, think about combining.

Conclusion

powerful and focused on taking action. Think about concluding with a succinct summary statement that highlights the findings' potential significance and urgency.

Reviewer 2

Name Kearney, Grainne

Affiliation Queen's University Belfast, Dentistry and Biomedical Sciences

Date 17-Nov-2025

COI None

Thank you for the opportunity to review this manuscript which I approach as an Academic GP and Educationalist. I believe it to be of importance and interest to the readership. It is carried out with rigour but I have some questions and concerns which if addressed, I believe, will add to the quality of the manuscript. The context section is helpful, and the PPIE aspect well executed.

In the binary choice above, I have hit 'no' for the two sections on how the question/objective is defined and the abstract - what I would have preferred to say is 'not yet'. - my next paragraph will suggest how I feel this can be modified.

For me, the main issue, from the title, abstract and aim, is the focus on GPRs as the group of interest but the work then goes onto look at qualified GPs also. There is mention that there isn't much literature around GPRs only so I think the case can be made to include inclusion of GPs but I don't think this is clearly spelt out at present. For me, this is the main issue that would benefit from being more clearly addressed.

Introduction

Within the introduction, I suggest there needs to be a statement around international provision of palliative care before focusing on the UK. My understanding is that the role of GPs in providing palliative care is not necessarily commonplace elsewhere, by Family Doctors internationally.

Methods

There is mention of inclusion of grey literature in the strengths and limitations, but I don't see than any grey literature was included. In fact the inclusion/exclusion criteria require peer review which seems contradictory.

I assume that literature was included if there as any mention of GPs i.e. even if alongside other colleagues? This only became clear when I saw the table on Pg 7. So like my earlier comment around inclusion of literature on GPs, I think this needs more clearly spelt out.

Small points

Pg 3 Line 58-9 doesn't make sense (sentence starting "Understanding caring …"

Pg 4 Line 50 –Family Practce

Results

Table 2 – I don't understand 'GPs to recall different stages of training'

Pg8 Line 52, the author used the word – physicians, was this to distinguish as doctors other than GPs?

The paragraph 'Readiness to care' I found difficult to follow, it appeared as a laundry list of literature. There seemed to be some signposting at the start but the writing then didn't follow this. Suggest this might be improved for the reader.

I was unsure of the title "Specific learning needs?", was this more "The skills and knowledge to consult"?

Pg10 Line 27, there is mention of dealing was a dying child, but this had been an exclusion.

Discussion

It might be helpful to take a more birds eye view of the data here. When you consider that the second title is about structural factors, it might be more intuitive to consider the 'Practice level factors' where you have used "Learning/emotional factors"

Small points

Pg 11 Line 56 prevent doctors from

Pg 11 Line 57 GP/GPRs

Pg 13 Line 11 – Sentence starting "Consistent opportunities …" is hard to follow

Pg 13 Line 37 changed

My understanding is that sometime the authors include the excel spreadsheet of included studies. I will defer to editor as to whether this would be helpful to include.

VERSION 1 - AUTHOR RESPONSE

Reviewer: 1	Thank you for all your comments and feedback. I have written responses to each point.
Abstract:<br>For clarity, think about dividing it into organized subheadings (Background, Objectives, Methods, Results, Conclusion).	Thank you. We have changed the separate methods subheadings to a more general 'methods'.
Introduction<br>For easier reading, several lengthy sentences might be divided.<br>For instance: "Offering excellent, comprehensive care to patients nearing the end of their lives in the community keeps patients in the setting of their choice and lowers the need for needless, expensive interventions and hospital stays." → For clarification, it might be divided into two sentences.	We have checked the wording and length of sentences throughout to ensure they are clear and divided if too long.
The phrases "end-of-life," "palliative," and "dying" are used interchangeably. Think about defining them in advance and using them consistently all the way through	The terms have been defined at the end of the introduction. It has been challenging to be consistent due to the many nuances which have been used interchangeably in the studies. This has been discussed as an unavoidable limitation of the scoping review.
Methods<br>It would be more accurate to say that "Ethical approval was not sought" instead of "Ethical approval was not sort."	This has been updated.
For improved reading, format the inclusion/exclusion table in the final version as a proper table.	We have changed the format of this table as suggested.
Make sure the additional appendices for search terms you mention is comprehensive and easily accessible.	We will add these in the supplementary files on final submission.
Results<br>Although it is not evident in the text, the PRISMA-ScR diagram is mentioned. Make sure the final submission contains it.	This is part of the main document but we have included it as a supplementary file as well for the final submission- thank you.
To make the four primary topics more visually identifiable, think about utilizing bullet points or subheadings.	We have changed to bullet points to show the main findings more clearly.
Table 2: For easy access, think about incorporating a summary table into the main text (rather than only supplementary).	Table 2 will be included in the main document as a table.
Discussion<br>The conversation is thorough and properly cited. Nonetheless, it would be advantageous to distinguish more clearly between: structural/systemic problems, learning/emotional variables, Consequences for training reform	Thank you for this comment. There is a lot of overlap in the literature between the discussion sub-heading areas which has made it difficult to separate the sections fully. The headings have been updated which helps distinguish the sections more.
Certain ideas are reiterated, such as the value of practical experience and mentorship. For conciseness, think about combining.	The discussion has been reviewed with small changes made throughout. Key findings have been reiterated due to their importance.
Conclusion<br>powerful and focused on taking action. Think about	This has been reviewed but no changes made due to word count restrictions.

concluding with a succinct summary statement that highlights the findings' potential significance and urgency.	
Reviewer: 2	Thank you for all your comments and feedback. We have written responses to each point.
For me, the main issue, from the title, abstract and aim, is the focus on GPRs as the group of interest but the work then goes onto look at qualified GPs also. There is mention that there isn't much literature around GPRs only so I think the case can be made to include inclusion of GPs but I don't think this is clearly spelt out at present. For me, this is the main issue that would benefit from being more clearly addressed.	Thank you for this comment, GPs have been included due to the lack of specific GPR literature but we agree this is not clear throughout. The title, abstract aim and focus has been adapted to be more transparent about the broadening of the original idea.
Introduction<br>Within the introduction, I suggest there needs to be a statement around international provision of palliative care before focusing on the UK. My understanding is that the role of GPs in providing palliative care is not necessarily commonplace elsewhere, by Family Doctors internationally.	This has been updated to reflect the international variation.
Methods<br>There is mention of inclusion of grey literature in the strengths and limitations, but I don't see than any grey literature was included. In fact the inclusion/exclusion criteria require peer review which seems contradictory.	Thank you for highlighting this, the article has been updated. Peer reviewed articles from the inclusion criteria has been removed as the search was broader than peer reviewed work. Grey literature was searched but there were no studies identified from this strategy.
I assume that literature was included if there as any mention of GPs i.e. even if alongside other colleagues? This only became clear when I saw the table on Pg 7. So like my earlier comment around inclusion of literature on GPs, I think this needs more clearly spelt out.	To make this clearer to the reader, we have changed the wording to include GPs throughout.
Pg 3 Line 58-9 doesn't make sense (sentence starting "Understanding caring	This sentence has been reworded for clarity.
Pg 4 Line 50 –Family Practce	Updated.
Table 2 – I don't understand 'GPs to recall different stages of training'	This has been reworded to make it clearer.
Pg8 Line 52, the author used the word – physicians, was this to distinguish as doctors other than GPs?	The wording for different doctor roles globally can be confusing. This survey asked primary care physicians who can be either internal medicine or family practice physicians, so this word is to include these doctor groups.
The paragraph 'Readiness to care' I found difficult to follow, it appeared as a laundry list of literature. There seemed to be some signposting at the start but the writing then didn't follow this. Suggest this might be improved for the reader.	We have changed the wording to improve readability.
I was unsure of the title "Specific learning needs?", was this more "The skills and knowledge to consult"?	Thank you for this suggestion, after much deliberation, we have decided to keep the original title as we feel it reflects the findings more.
Pg10 Line 27, there is mention of dealing was a dying child, but this had been an exclusion.	This statement has been removed.
Discussion<br>It might be helpful to take a more birds eye view of the data here. When you consider that the second title is about structural factors, it might be more intuitive to consider the 'Practice level factors' where you have used "Learning/emotional factors"	This has been reviewed and updated to include practice level factors.
Pg 11 Line 56 prevent doctors from	changed
Pg 11 Line 57 GP/GPRs	changed
Pg 13 Line 11 – Sentence starting "Consistent opportunities …" is hard to follow	This sentence has been updated.
Pg 13 Line 37 changed	Changed
My understanding is that sometime the authors include the excel spreadsheet of included studies. I will defer to editor as to whether this would be helpful to include.	Thank you for highlighting this. We have made the changes suggested by the editor that agrees with your comment.

VERSION 2 - REVIEW

**Reviewer**	**2**
**Name**	**Kearney, Grainne**
**Affiliation**	**Queen's University Belfast, Dentistry and Biomedical Sciences**
**Date**	**07-Dec-2025**
**COI**	

Thank you for addressing my points so comprehensibly. I look forward to seeing this in print

VERSION 2 - AUTHOR RESPONSE

Reviewer: 2<br>Dr. Grainne Kearney, Queen's University Belfast<br>Comments to the Author:<br>Thank you for addressing my points so comprehensibly. I look forward to seeing this in print<br>Reviewer: 2<br>If you have selected 'Yes' above, please provide details of any competing interests.: Not applicable	Thank you very much for reviewing the article and your valuable suggestions and comments.
